# Fate predetermination of cardiac myocytes during zebrafish heart regeneration

**DOI:** 10.1098/rsob.170116

**Published:** 2017-06-28

**Authors:** Isil Tekeli, Anna Garcia-Puig, Mario Notari, Cristina García-Pastor, Isabelle Aujard, Ludovic Jullien, Angel Raya

**Affiliations:** 1 Center of Regenerative Medicine in Barcelona (CMRB), Hospital Duran i Reynals, Hospitalet de Llobregat (Barcelona), 3rd Floor, Avinguda de la Gran Via 199-203, 08908 Barcelona, Spain; 2 Center for Networked Biomedical Research on Bioengineering, Biomaterials and Nanomedicine (CIBER-BBN), Madrid, Spain; 3 Department of Chemistry, École Normale Supérieure—PSL Research University, 24 rue Lhomond, 75005 Paris, France; 4 Sorbonne Universités, UPMC Univ Paris 06, PASTEUR, 75005 Paris, France; 5 CNRS, UMR 8640 PASTEUR, 75005 Paris, France; 6 Institució Catalana de Recerca i Estudis Avançats (ICREA), Barcelona, Spain

**Keywords:** cardiomyocytes, lineage-tracing, cell migration, heart development, Cre/lox recombination

## Abstract

Adult zebrafish have the remarkable ability to regenerate their heart upon injury, a process that involves limited dedifferentiation and proliferation of spared cardiomyocytes (CMs), and migration of their progeny. During regeneration, proliferating CMs are detected throughout the myocardium, including areas distant to the injury site, but whether all of them are able to contribute to the regenerated tissue remains unknown. Here, we developed a CM-specific, photoinducible genetic labelling system, and show that CMs labelled in embryonic hearts survive and contribute to all three (primordial, trabecular and cortical) layers of the adult zebrafish heart. Next, using this system to investigate the fate of CMs from different parts of the myocardium during regeneration, we show that only CMs immediately adjacent to the injury site contributed to the regenerated tissue. Finally, our results show an extensive predetermination of CM fate during adult heart regeneration, with cells from each myocardial layer giving rise to cells that retain their layer identity in the regenerated myocardium. Overall, our results indicate that adult heart regeneration in the zebrafish is a rather static process governed by short-range signals, in contrast to the highly dynamic plasticity of CM fates that takes place during embryonic heart regeneration.

## 1. Introduction

The zebrafish has positioned itself as a valuable model for investigating heart regeneration due to its intrinsic capacity to restore large portions of this organ after amputation [1,2], cryoinjury [3–5] or genetic ablation [6]. Following damage of up to 20% of the heart ventricle, adult zebrafish can fully regenerate it through multiple mechanisms that involve limited dedifferentiation of spared cardiomyocytes (CMs), sarcomere disassembly, and CM proliferation through the re-expression of cell cycle regulators such as *plk1* and *mps1*. As demonstrated by lineage-tracing approaches, newly generated CMs arise from pre-existing ones, and therefore CM proliferation appears to be the primary mechanism for CM regeneration after amputation or injury [7,8]. Cell proliferation assays such as 5-bromo-2′-deoxyuridine (BrdU) or 5-ethynyl-2′-deoxyuridine (EdU) incorporation have shown that most proliferating CMs are located close to the injury site (between 65 and 75%), although some can also be found throughout the entire myocardium (from 10 up to 30%) [7,9,10]. Whether proliferating CMs located distant to the injury site actually contribute to myocardial regeneration is currently unknown. However, two pieces of indirect evidence support that this might be the case. First, in the adult zebrafish, active migration of CMs towards the injury site was shown to be essential for heart regeneration [9]. Second, in zebrafish larvae, CMs from the atrium transdifferentiated into ventricular CMs and migrated all the way to the ventricle, where they contributed to myocardial regeneration [11].

At any rate, it should be noted that this type of cell proliferation assay provides information as to the location of BrdU- or EdU-labelled cells at the time of analysis, which may be different from their original position when they began proliferating and incorporated BrdU/EdU. This raises the intriguing possibility that CMs could proliferate in specific, perhaps distant zones of the heart, and migrate towards the regenerating area where they would contribute to the newly formed myocardium. An analogous situation has been long known in the context of lens regeneration in newts, where only the pigmented epithelial cells from the dorsal iris contribute to the regenerative process by transdifferentiating into lens cells [12]. By contrast, planarian neoblasts are absent in the most anterior part of the head and the pharynx, making these regions regeneration-incompetent [13]. The existence of such regeneration-competent (or regeneration-incompetent) areas has not been previously explored in the context of zebrafish heart regeneration. In this study, we sought to address these two outstanding questions: whether the CMs that proliferate at places distant to the injury site actually contribute to the regenerated myocardium, and if so, whether they are distributed randomly throughout the zebrafish heart, or they make up specific, regeneration-competent areas.

For this purpose, we have developed a genetic lineage-tracing strategy based on the conditional expression of a fluorescent reporter transgene in few CMs by *Cre/lox* recombination. As this genetic modification is permanent and inherited by the progeny of the labelled CMs, this system allows lineage tracing of labelled CMs during development and regeneration. Our results show that CMs display a short-range migration, and only CMs immediately adjacent to the injury site contribute to the regenerating myocardium.

## 2. Material and methods

### 2.1. Zebrafish maintenance and surgical procedures

AB strain wild-type and transgenic zebrafish were maintained and raised according to standard protocols [14]. The generation and characterization of the transgenic zebrafish lines Tg(myl7:Cre-Ert2)^+/−^ and Tg(myl7:LnL-EGFP)^+/−^ were described previously [7]. The ventricular amputations were done according to the published protocol [2].

### 2.2. Application of cyclofen and caged-cyclofen

Cyclofen and caged-cyclofen were synthesized and prepared as described previously [15]. Zebrafish embryos were incubated overnight in the embryo medium containing 2 µM caged-cyclofen prior to UV exposure. Before UV irradiation, the embryos were washed with fresh embryo medium to avoid any possible interference, which could originate from photoreleasing the caged-cyclofen in the medium.

### 2.3. Ultraviolet illumination

Zebrafish embryos were treated with 75 µM 1-phenyl 2-thiourea (PTU) at 22 hpf (28 somite stage) as previously described [16], in order to inhibit pigmentation and allow UV light to reach the heart tissue. Prior to UV illumination, 2 day post-fertilization (dpf) zebrafish were anaesthetized using 4.2 ml tricaine solution (4 mg ml^−1^) per 100 ml of fish tank water [14]. UV illumination was achieved using a bench-top UV lamp (Spectroline FC-100) that emits light of wavelength 365 nm, for 4 min at a distance of 10 cm from the working surface. Screening for positive or negative results was done 1 and 2 days following each experiment.

### 2.4. Immunofluorescence analyses

Hearts were fixed overnight in 4% paraformaldehyde at 4°C, washed with PBS several times, equilibrated in 30% sucrose in PBS and frozen in OCT (Tissue-Tek) for cryosectioning. Immunohistochemistry was performed on 10 µm cryosections by using MF20 (1 : 1, DSHB), anti-BrdU (1 : 50, Accurate) and anti-GFP (1 : 200, GFP-1020; Aves Labs) primary antibodies; and DyLight 488 (1 : 50, Jackson Immuno Research Laboratories), Cy5 (1 : 200, Jackson Immuno Research Laboratories) and Cy3 (1 : 200, Jackson Immuno Research Laboratories) as secondary antibodies.

### 2.5. Microscopy, *in vivo* imaging and image processing

General screening of the zebrafish larvae for GFP-positive labelled CMs was done under a stereomicroscope (Leica). Detailed images of the positive larvae were obtained using a spinning-disk confocal microscopy system (PerkinElmer UltraViewERS Spinning-Disk) mounted on a Zeiss Axiovert 200M microscope equipped with a Hamamatsu C9100-50 EMCCD camera. Whole-mount hearts and the heart sections were imaged using a stereomicroscope (Leica), and a confocal microscope (Leica SP5), respectively. Images were processed with Adobe Photoshop.

## 3. Results and discussion

### 3.1. Genetic labelling of zebrafish cardiomyocytes

To label different ventricular regions of the zebrafish heart, we developed a myocardium-specific UV-inducible Cre/*lox* recombination system. In this system, CM labelling is based on green fluorescent protein (GFP) expression only in the CMs of transgenic zebrafish upon Cre recombination. For this, we first generated a double transgenic zebrafish line by crossing Tg(myl7:Cre-Ert2)^+/−^ and Tg(myl7:LnL-EGFP)^+/−^ [7] (electronic supplementary material, figure S1). In these transgenic fish, GFP is expressed only in CMs when a recombination event is induced by tamoxifen administration. In order to label CMs in a specific area, we took advantage of cyclofen, a synthetic analogue of tamoxifen, which can be synthesized as inactive (caged-cyclofen) and turned into its active form by freeing it from its caging group upon UV illumination [15]. This feature of the system allows *Cre/lox* recombination to take place only in the cells that are photoactivated.

**Figure 1.**
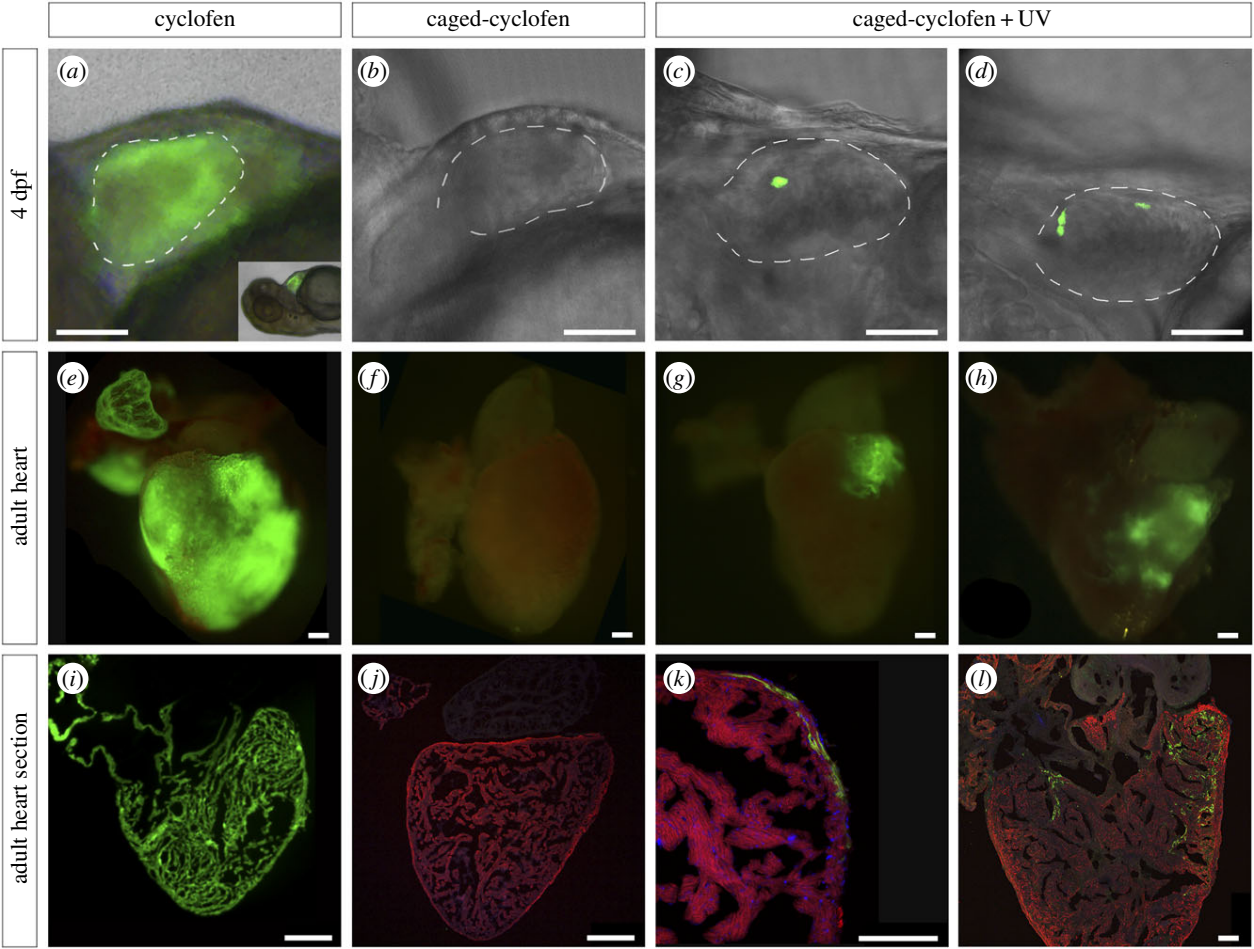
Genetic labelling of zebrafish CMs using a UV-inducible Cre/*lox* recombination system. Double transgenic embryos (Tg(myl7:Cre-Ert2)^+/−^ × Tg(myl7:LnL-EGFP)^+/−^) were treated at 2 dpf with (*a*) uncaged cyclofen (*n* = ∼100), (*b*) caged-cyclofen without UV exposure (*n* = ∼100) and (*c,d*) caged-cyclofen followed by UV exposure (*n* = ∼100). One fish tank per condition (approx. 40 larvae) was raised to adulthood. Cyclofen works as a tamoxifen analogue and induces recombination in CMs as observed by GFP expression at 4 dpf (*a*) and at adult stage (*e,i*). (*b,f,j*) Caged-cyclofen cannot induce recombination unless it is uncaged by UV exposure (*c*,*d*,*g*,*h*,*k*,*l*). Adult heart sections were processed for immunofluorescence with antibodies against MF20 and GFP. Nuclei were counterstained with DAPI. Scale bars: (*a–d*) 150 µm, (*e–j*) 250 µm, (*k–l*) 100 µm. Dashed lines in (*a*–*d*) define the ventricle perimeter.

To test whether cyclofen was able to induce Cre/*lox* recombination in our system, we treated 2 dpf embryos with uncaged cyclofen and assessed GFP expression 2 days later. After confirming that the recombination occurred successfully and specifically in CMs (figure 1*a*), these embryos were raised to adulthood to verify that GFP expression was permanent (figure 1*e,i*). To test the existence of leakage in our system, we treated 2 dpf embryos with caged-cyclofen and did not expose them to UV light. In those hearts, no recombination events were induced, as judged by the absence of GFP expression at 4 dpf (*n* ∼ 100) (figure 1*b*), and in adult animals (*n* = 30) (figure 1*f,j*). By contrast, we observed successful recombination and strong GFP expression in confined areas of embryonic zebrafish hearts upon UV illumination (figure 1*c,d*). By optimizing the time of illumination and distance from the UV light source, we could obtain hearts with small GFP-labelled areas of an average diameter size of 21.8 ± 3.1 µm (*n* = 7) [17], indicating that each GFP-positive area comprised 1–2 adjacent CMs (electronic supplementary material, figure S2). As expected, these labelled embryos maintained GFP expression in their hearts as adults, demonstrating that labelled CMs survived and passed the recombined construct onto their progeny (figure 1*g,h,k,l*). So far, these results show that we are able to label a small group of adjacent CMs in the embryonic zebrafish heart for further lineage tracing analysis during development and regeneration by using this approach. Unlike three-photon activation used to label single cells [17], a non-directed activation was used in this study to label many different areas throughout the ventricle to study the contribution of these labelled CMs to regeneration and be able to create a map of regeneration competent or incompetent areas of the adult zebrafish ventricle.

### 3.2. Genetically labelled cardiomyocytes were found in primordial, trabecular and cortical layers

**Figure 2.**
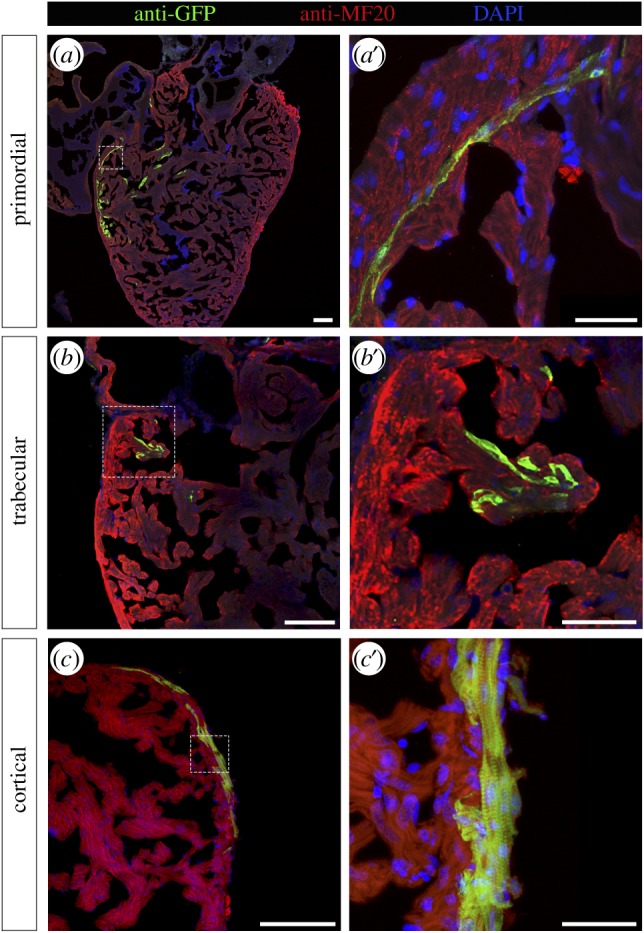
Genetically labelled CMs are found in all three myocardium layers. Adult zebrafish hearts labelled at 2 dpf were sectioned and processed for immunofluorescence with antibodies against MF20 and GFP. GFP-labelled CMs were identified in (*a*,*a*′) the primordial layer, (*b*,*b*′) the trabecular layer and (*c*,*c*′) the cortical layer. Nuclei were counterstained with DAPI. Scale bars: (*a–c*) 100 µm, (*a*′–*c*′) 25 µm. Dashed lines in (*a*–*c*) define the magnification areas shown in (*a*′–*c*′), respectively.

The traditional view of the zebrafish ventricle consisting of two types of cardiac muscle, a peripheral wall of compact muscle and inner trabecular muscle, has been revised by the observation of primordial and cortical muscle lineages within the compact layer [18]. Analyses of GFP-positive areas in 3-month-old adult hearts labelled as embryos with our novel genetic system provided further evidence for their existence. Thus, we could observe GFP-positive CMs in the innermost part of the compact myocardium forming a one-cell thick layer (figure 2*a*,*a*′), highly reminiscent of the primordial layer described by Gupta & Poss [18]. Surprisingly, this structure was never found labelled alone in our labelled hearts, but always accompanied by labelled CMs in the trabecular zone (*n* = 16), suggesting that primordial CMs gave rise to trabecular CMs in all of the hearts analysed. Additionally, we could detect GFP-positive areas located in the outer part of the compact myocardium in adult hearts (figure 2*c*,*c*′), which would correspond to the cortical layer [18]. Therefore, our results provide independent confirmation of the visualization of three myocardial layers (primordial, trabecular and cortical) in the zebrafish heart, using a direct lineage tracing system, and demonstrate that CMs labelled in embryonic hearts survive and contribute to all three layers in adult myocardium.

Out of 30 hearts that were successfully labelled at 2 dpf and verified to contain GFP-positive CMs at 4 dpf, when analysed as adults, 23 hearts still maintained GFP-labelled areas (electronic supplementary material, table S1). The absence of GFP-positive CMs in the remaining seven hearts indicates that either labelled embryonic cells died before giving rise to progeny or they did not produce progeny at all. This is consistent with previous studies showing that from around 115 embryonic CMs, only around 55 (roughly 50%) go on to form the juvenile zebrafish myocardium [18]. Among the adult hearts that maintained GFP-labelled areas, approximately one-third (seven out of 23, 30%) showed labelled CMs distributed along the three myocardial layers, whereas 40% (nine out of 23) occupied both primordial and trabecular layers, and the remaining 30% (seven out of 23) of GFP-positive areas contained exclusively trabecular CMs (electronic supplementary material, table S1). It is interesting to note that all labelled areas in adult hearts comprised trabecular muscle, even when no GFP-positive CMs were found in the primordial layer. Previous studies have suggested that the trabecular heart muscle originates from primordial CMs, which detach from the myocardial wall and then reattach, sometimes even at distant places, giving rise to trabeculae [18–20]. The relative distribution of GFP-labelled areas found in our studies is consistent with this scenario, and further points out that most primordial CMs should be able to give rise to trabecular muscle, even though they do not contribute to the adult primordial layer. Finally, the GFP-positive areas comprising cortical myocardium detected in our studies ranged in size from 1.2 to 11% of the ventricular surface (electronic supplementary material, figure S3), indicating that none of them had labelled a dominant clone in agreement with their reported low frequency [18].

### 3.3. Short-range migration of cardiomyocytes during adult heart regeneration

**Figure 3.**
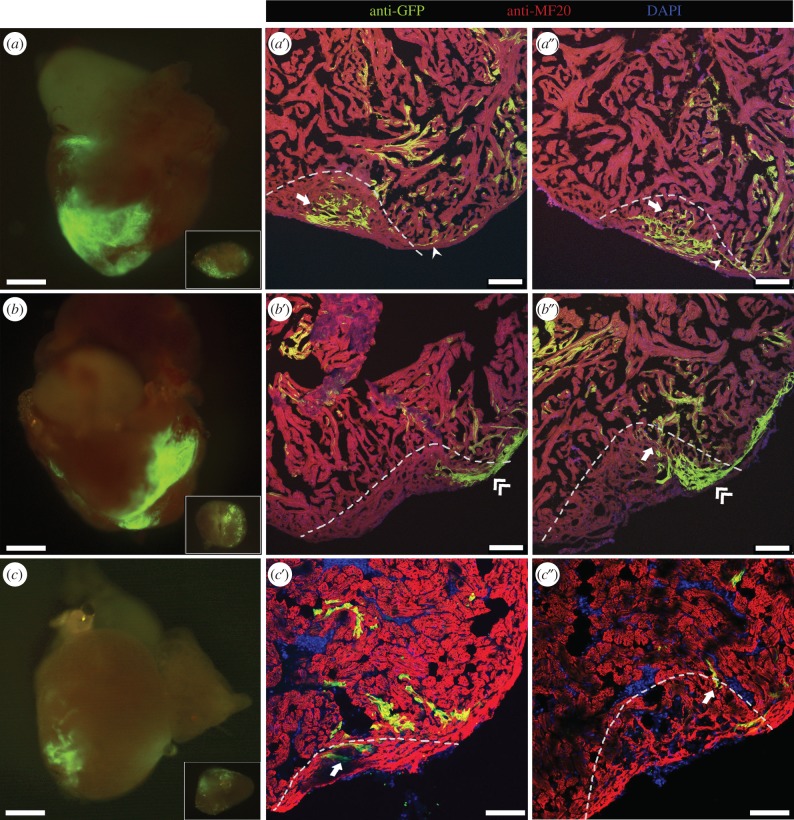
Cell fate of labelled CMs during regeneration. Three-month-old adult zebrafish labelled at 2 dpf were subjected to ventricular amputation, and after 30 dpa the hearts were collected. (*a–c*) Epifluorescence images of representative hearts where the amputation plane passed through GFP-positive areas. The excised portion of tissue is shown in inset. (*a*′–*c*″) Collected hearts were sectioned, and processed for immunofluorescence with antibodies against MF20 and GFP. (*a*′,*a″*) Different sections of the regenerated myocardium of the heart shown in (*a*), where primordial (arrowhead) and trabecular (arrow) labelled CMs were observed. (*b*′,*b*″) Different sections of the regenerated myocardium of the heart shown in (*b*), where trabecular (arrow) and cortical (double-arrowhead) GFP-positive CMs were detected. (*c*′,*c″*) Different sections of the regenerated myocardium of the heart shown in (*c*), where trabecular (arrow) GFP-positive CMs were detected. The amputation plane is indicated by dashed lines. Nuclei were counterstained with DAPI. Scale bars: 500 µm in whole-mount hearts; 100 µm in sections.

To analyse the contribution of different GFP-labelled areas to regeneration, we amputated the ventricular apex of 3-month-old fish that had been labelled at 2 dpf and verified to contain GFP-positive CMs at 4 dpf (*n* = 24). We then analysed the presence of GFP-labelled CMs in the regenerated tissue at 30 days post-amputation (dpa), a time when the regenerative process is well underway and most de novo CMs have been produced [1,2]. In five of the 24 hearts processed and analysed in this way, the amputation plane passed through a GFP-labelled area, as shown by the presence of GFP-positive cells in the piece of excised apex tissue (figure 3*a–c*). In two of these five cases, the GFP-labelled areas affected by the amputation comprised trabecular and primordial myocardial layers, and the regenerated tissue after 30 dpa also contained GFP-positive CMs in the same layers (figure 3*a*′,*a″*). Similarly, the amputation plane cut through labelled trabecular and cortical layers in one heart, and the regenerated tissue contained labelled CMs from these two layers (figure 3*b*′,*b*″). Moreover, in two of these five cases, when the amputation plane comprised only trabecular layer, the regenerated tissue contained labelled CMs only in this layer (figure 3*c*′,*c*″). In all cases, the seamless continuation of labelled cells from the spared myocardium into the regenerated tissue indicates that CMs in the primordial, trabecular and cortical layers give rise to new CMs in the corresponding layers of the regenerated myocardium.

**Figure 4.**
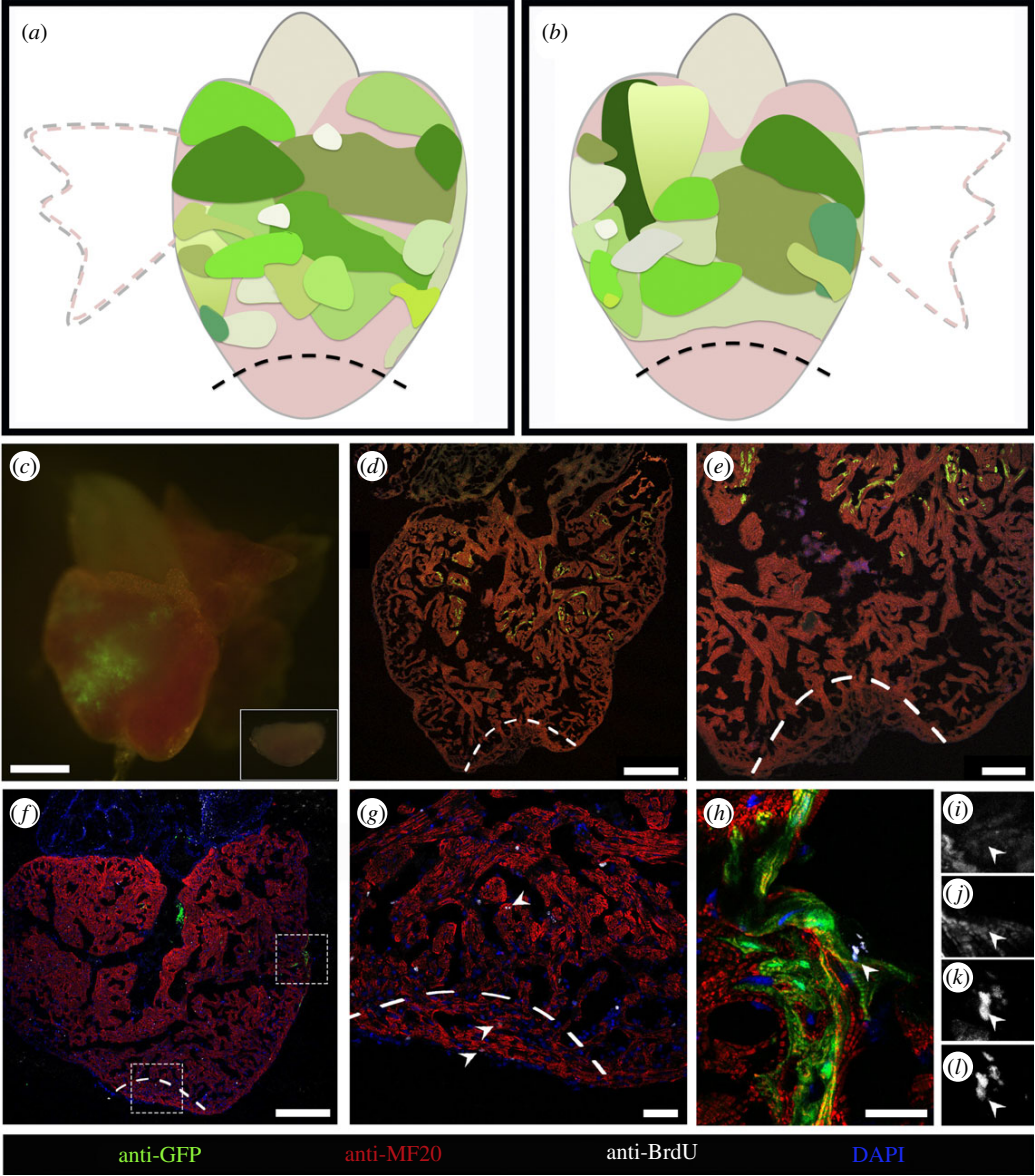
CMs distant from the amputation site do not contribute to the regenerated tissue. (*a,b*) Diagram summarizing the size and location of GFP-positive areas from different hearts which did not contribute to regeneration (*n* = 15). (*a*) The dorsal view of a zebrafish heart and (*b*) the ventral view. (*c*) Epifluorescence image of a representative heart where the amputation plane did not pass through the GFP-positive area. The excised portion of tissue is shown in inset. (*d,e*) Collected hearts were sectioned, and processed for immunofluorescence with antibodies against MHC and GFP. GFP-labelled CMs are absent in the regenerated area. (*f–h*) Sections processed for immunofluorescence with antibodies against BrdU, GFP and MHC showing the proliferating CMs (arrowheads) in the injury site (*g*) and in the GFP-positive area (*h*). Single image channels of the BrdU-positive GFP-positive CM (arrowhead) shown in (*h*), where (*i*) is GFP, (*j*) is MHC, (*k*) is DAPI and (*l*) is BrdU. Magnification images of lower and upper dashed rectangles in (*f*) are shown in (*g*) and (*h*), respectively. The amputation plane is indicated by dashed lines. Nuclei were counterstained with DAPI. Scale bars: (*c*) 500 µm, (*d,f*) 250 µm, (*e*) 100 µm, (*g*) 40 µm and (*h*)15 µm.

With the aim of investigating the existence of specific regeneration-competent areas within the myocardium, and whether the migration of CMs from such zones towards the injury site would occur during regeneration, we performed ventricular amputation in 19 adult zebrafish in which the GFP-labelled areas were located away from the apex. Taken together, the GFP-labelled areas in these hearts covered the vast majority of the ventricular surface, but the amputation plane did not cut through them as also judged by the absence of GFP signal in the resected portion of the ventricles (figure 4*a,b*; electronic supplementary material, figure S4). When we analysed the regenerated area at 30 dpa, we could not find GFP-labelled CMs in any of these hearts (figure 4*c–e*). Thus, our results indicate that CMs proliferating at a distance from the regenerating area, which were found in several GFP-labelled clones (figure 4*f,h–l*; electronic supplementary material, figure S5), do not contribute to the regenerated myocardium in comparison to proliferating CMs in the regenerating area (figure 4*g*). CM proliferation accounts for the regular haemostasis of the zebrafish heart [21], so it is likely that the proliferating CMs that we show do not contribute to the regenerated tissue could instead serve to provide higher mechanical force to compensate for the lost tissue. In any case, our combined findings strongly argue against the existence of specific regeneration-competent areas in the adult zebrafish myocardium.

The present studies take advantage of the direct lineage tracing of CMs during development and regeneration, made possible thanks to a novel myocardium-specific, photoinducible genetic labelling system. Lineage tracing of CMs during development provides independent confirmation of the presence of primordial, trabecular and cortical layers in the adult zebrafish heart by using a technique complementary to the one used in the original description of these layers [18]. Cell tracing during adult heart regeneration showed that CMs adjacent to the injury site gave rise to the CMs in the regenerated myocardium, whereas those located farther away, even though they may undergo cell proliferation in response to the injury, did not contribute to the regenerated tissue. These results are in agreement with recent findings showing that 50% of mitotic CMs are found within the first 100 µm adjacent to the injury zone at 7 dpa [10]. The overall picture that emerges from our studies of direct CM lineage tracing during adult heart regeneration is of a rather static process, in sharp contrast to the highly dynamic CM fate reprogramming described to take place during embryonic heart regeneration [11]. According to our results, only CMs very close to the injury site would be able to proliferate, migrate (perhaps through a cxcl12a-cxcr4b-dependent mechanism) [9] and give rise to new CMs in the regenerated myocardium. Furthermore, our results show that the predetermination of CM fate during adult heart regeneration is so prevailing that even their identity of layer of origin is retained, and subsequently reproduced in the regenerated tissue. Our combined results reveal important differences in the cellular bases (and probably molecular mechanisms as well) that underlie the process of heart regeneration in developing embryos and in the adult setting, and highlight the key role that short-range signals should have for controlling the latter.

## Supplementary Material

Supplementary Information

## Data accessibility

The datasets supporting this article have been uploaded as part of the electronic supplementary material.

## References

[1] Poss KD, Wilson LG, Keating MT 2002 Heart regeneration in zebrafish. Science 298, 2188–2190. (doi:10.1126/science.1077857) 12481136 10.1126/science.1077857

[2] Raya A et al. 2003 Activation of notch signaling pathway precedes heart regeneration in zebrafish. Proc. Natl Acad. Sci. USA 100(Suppl. 1), 11 889–11 895. (doi:10.1073/pnas.1834204100) 10.1073/pnas.1834204100 PMC304103 12909711

[3] Chablais F, Veit J, Rainer G, Jazwinska A 2011 The zebrafish heart regenerates after cryoinjury-induced myocardial infarction. BMC Dev. Biol. 11, 21 (doi:10.1186/1471-213X-11-21) 21473762 10.1186/1471-213X-11-21 PMC3078894

[4] Gonzalez-Rosa JM, Martin V, Peralta M, Torres M, Mercader N 2011 Extensive scar formation and regression during heart regeneration after cryoinjury in zebrafish. Development 138, 1663–1674. (doi:10.1242/dev.060897) 21429987 10.1242/dev.060897

[5] Schnabel K, Wu CC, Kurth T, Weidinger G 2011 Regeneration of cryoinjury induced necrotic heart lesions in zebrafish is associated with epicardial activation and cardiomyocyte proliferation. PLoS ONE 6, e18503 (doi:10.1371/journal.pone.0018503) 21533269 10.1371/journal.pone.0018503 PMC3075262

[6] Wang J et al. 2011 The regenerative capacity of zebrafish reverses cardiac failure caused by genetic cardiomyocyte depletion. Development 138, 3421–3430. (doi:10.1242/dev.068601) 21752928 10.1242/dev.068601 PMC3143562

[7] Jopling C, Sleep E, Raya M, Marti M, Raya A, Izpisua Belmonte JC 2010 Zebrafish heart regeneration occurs by cardiomyocyte dedifferentiation and proliferation. Nature 464, 606–609. (doi:10.1038/nature08899) 20336145 10.1038/nature08899 PMC2846535

[8] Kikuchi K et al. 2010 Primary contribution to zebrafish heart regeneration by *gata4*^+^ cardiomyocytes. Nature 464, 601–605. (doi:10.1038/nature08804) 20336144 10.1038/nature08804 PMC3040215

[9] Itou J, Oishi I, Kawakami H, Glass TJ, Richter J, Johnson A, Lund TC, Kawakami Y 2012 Migration of cardiomyocytes is essential for heart regeneration in zebrafish. Development 139, 4133–4142. (doi:10.1242/dev.079756) 23034636 10.1242/dev.079756

[10] Sallin P, de Preux Charles AS, Duruz V, Pfefferli C, Jazwinska A 2015 A dual epimorphic and compensatory mode of heart regeneration in zebrafish. Dev. Biol. 399, 27–40. (doi:10.1016/j.ydbio.2014.12.002) 25557620 10.1016/j.ydbio.2014.12.002

[11] Zhang R et al. 2013 In vivo cardiac reprogramming contributes to zebrafish heart regeneration. Nature 498, 497–501. (doi:10.1038/nature12322) 23783515 10.1038/nature12322 PMC4090927

[12] Eguchi G, Shingai R 1971 Cellular analysis on localization of lens forming potency in the newt iris epithelium. Dev. Growth Differ. 13, 337–349. 5152591 10.1111/j.1440-169x.1971.00337.x

[13] Salvetti A, Rossi L, Bonuccelli L, Lena A, Pugliesi C, Rainaldi G, Evangelista M, Gremigni V 2009 Adult stem cell plasticity: neoblast repopulation in non-lethally irradiated planarians. Dev. Biol. 328, 305–314. (doi:10.1016/j.ydbio.2009.01.029) 19389358 10.1016/j.ydbio.2009.01.029

[14] Westerfield M 2000 The zebrafish book: a guide for the laboratory use of zebrafish (Danio rerio). Eugene, OR: University of Oregon Press.

[15] Sinha DK et al. 2010 Photocontrol of protein activity in cultured cells and zebrafish with one- and two-photon illumination. Chembiochem 11, 653–663. (doi:10.1002/cbic.201000008) 20187057 10.1002/cbic.201000008

[16] Karlsson J, von Hofsten J, Olsson PE 2001 Generating transparent zebrafish: a refined method to improve detection of gene expression during embryonic development. Mar. Biotechnol. (NY) 3, 522–527. (doi:10.1007/s1012601-0053-4) 14961324 10.1007/s1012601-0053-4

[17] Tekeli I, Aujard I, Trepat X, Jullien L, Raya A, Zalvidea D 2016 Long-term in vivo single-cell lineage tracing of deep structures using three-photon activation. Light Sci. Appl. 5, e16084 (doi:10.1038/lsa.2016.84) 10.1038/lsa.2016.84 PMC6059956 30167169

[18] Gupta V, Poss KD 2012 Clonally dominant cardiomyocytes direct heart morphogenesis. Nature 484, 479–484. (doi:10.1038/nature11045) 22538609 10.1038/nature11045 PMC3340018

[19] Liu J, Bressan M, Hassel D, Huisken J, Staudt D, Kikuchi K, Poss KD, Mikawa T, Stainier DY 2010 A dual role for ErbB2 signaling in cardiac trabeculation. Development 137, 3867–3875. (doi:10.1242/dev.053736) 20978078 10.1242/dev.053736 PMC3049280

[20] Staudt DW, Liu J, Thorn KS, Stuurman N, Liebling M, Stainier DY 2014 High-resolution imaging of cardiomyocyte behavior reveals two distinct steps in ventricular trabeculation. Development 141, 585–593. (doi:10.1242/dev.098632) 24401373 10.1242/dev.098632 PMC3899815

[21] Wills AA, Holdway JE, Major RJ, Poss KD 2008 Regulated addition of new myocardial and epicardial cells fosters homeostatic cardiac growth and maintenance in adult zebrafish. Development 135, 183–192. (doi:10.1242/dev.010363) 18045840 10.1242/dev.010363

