# Supplementary material for: Fate predetermination of cardiac myocytes during zebrafish heart regeneration
Source: Open Biol. 2017 Jun 28;7(6):170116. doi: 10.1098/rsob.170116 (PMC5493782; doi:10.1098/rsob.170116)
Supplement: Supplementary Information [file rsob170116supp1.docx]

**Electronic Supplementary Material**

**Fate predetermination of cardiac myocytes during zebrafish heart regeneration**

Isil Tekeli^1^, Anna Garcia-Puig^1^, Mario Notari^1,2^, Cristina García-Pastor^1,2^, Isabelle Aujard^3,4,5^, Ludovic Jullien^3,4,5^, Angel Raya^1,2,6^*

^1^ Center of Regenerative Medicine in Barcelona (CMRB), Barcelona Biomedical Research Park, Dr. Aiguader 88, 08003 Barcelona, Spain.

^2^ Center for Networked Biomedical Research on Bioengineering, Biomaterials and Nanomedicine (CIBER-BBN).

^3^ École Normale Supérieure – PSL Research University, Department of Chemistry, 24 rue Lhomond, F-75005 Paris, France.

^4^ Sorbonne Universités, UPMC Univ Paris 06, PASTEUR, F-75005 Paris, France.

^5^ CNRS, UMR 8640 PASTEUR, F-75005 Paris, France.

^6^ Institució Catalana de Recerca i Estudis Avançats (ICREA).

| **Labeled myocardium layer** | **Number of hearts** |
| --- | --- |
| No label | 7 |
| Primordial only (P) | 0 |
| Trabecular only (T) | 7 |
| Cortical only (C) | 0 |
| P + T | 9 |
| P + C | 0 |
| T + C | 0 |
| P + T + C | 7 |
| **TOTAL** | **30** |

**Supplementary Table S1. Distribution of the myocardium layers within the labeled areas of the adult hearts** (n=30). GFP-labeled areas were composed of only trabecular layer (n=7), primordial and trabecular layer together (n=9), and all three layers together (n=7). 7 of the 2dpf-labeled hearts were not GFP-labeled by adulthood (~3 months old). dpf, days post fertilization

**
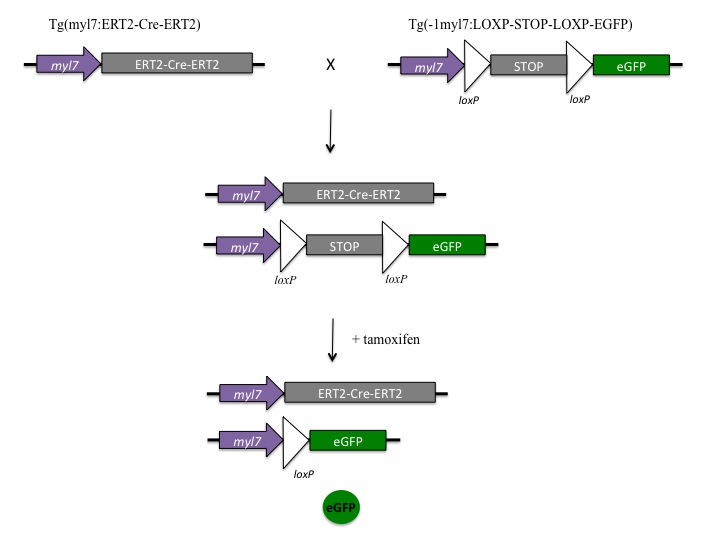
Supplementary Fig. S1**. **Cre/lox recombination-based genetic labeling system**. A double transgenic line was created by crossing following transgenic lines: (**1**) Tg(myl7:ERT2-Cre-ERT2) in which Cre recombinase is expressed only in the cardiomyocytes under the cardiomyocyte specific promoter myl7, and (**2**) Tg(myl7:LoxP-STOP-LoxP-EGFP) in which GFP sequence is placed after a floxed stop sequence under myl7 promoter. In the double transgenic fish, Cre recombinase becomes functional upon tamoxifen administration and excises the stop sequence, enabling GFP expression only in the cardiomyocytes.

**
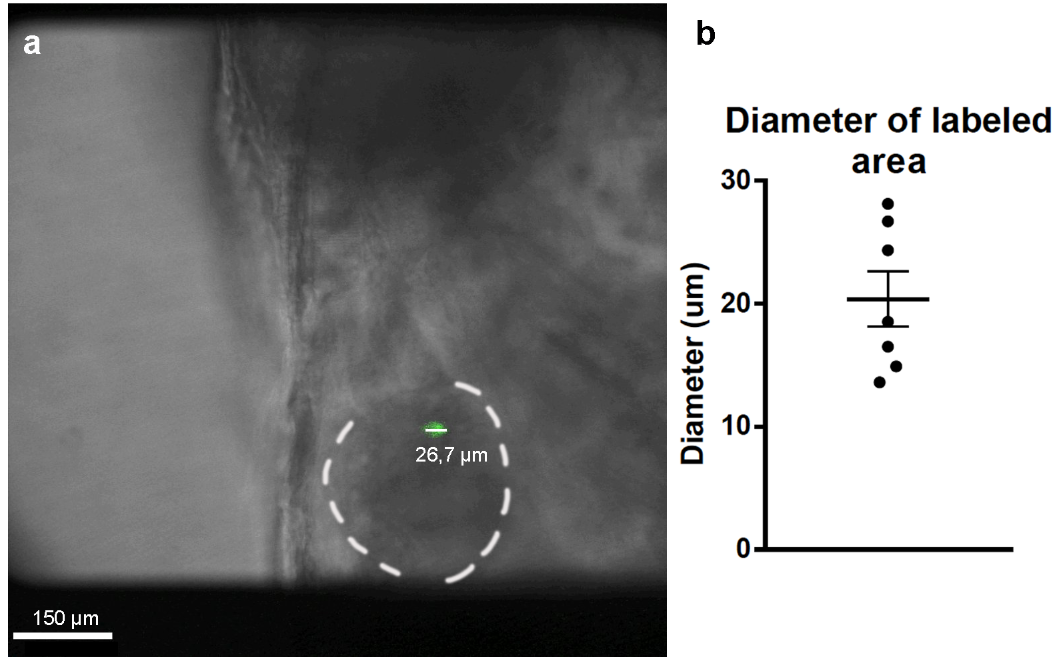
**

**Supplementary Fig. S2**. **Diameters of the labeled areas in 4dpf zebrafish hearts**. (**a**) Images of the labeled hearts were taken at 4 dpf and the diameters or the GFP-labeled areas were measured using ImageJ. (**b**) Measured diameters indicated that these labeled areas corresponded to one or two cardiomyocytes (n=7). Mean ± SEM.

**
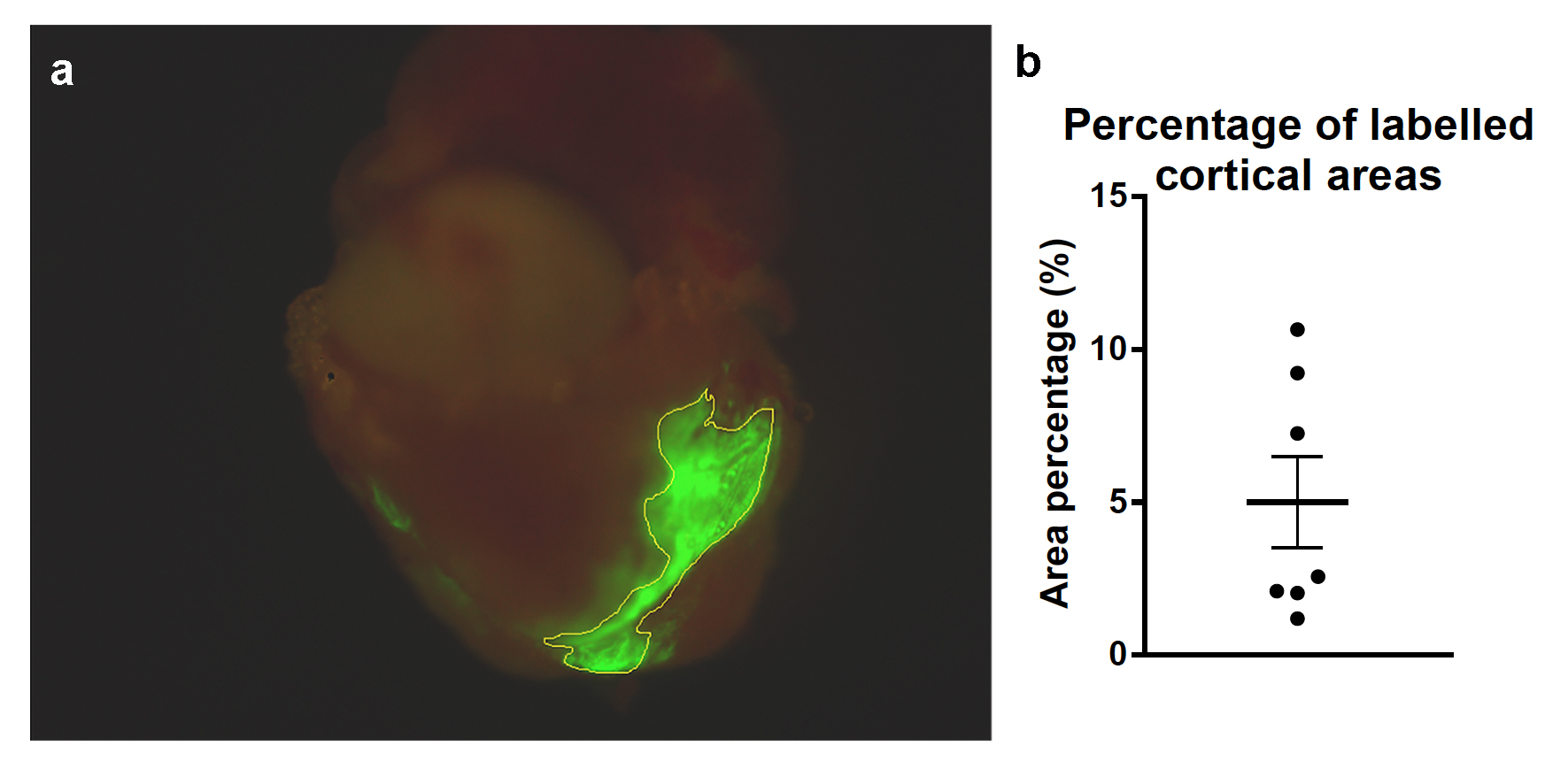
**

**Supplementary Fig. S3**. **Labeled cortical area size**. (**a**) Percentages of the cortical areas were calculated from the images of whole-mount hearts by using ImageJ. (**b**) Individual values obtained for 7 labeled cortical areas. Calculated areas ranged from 1.2% to 11%. Mean ± SEM.


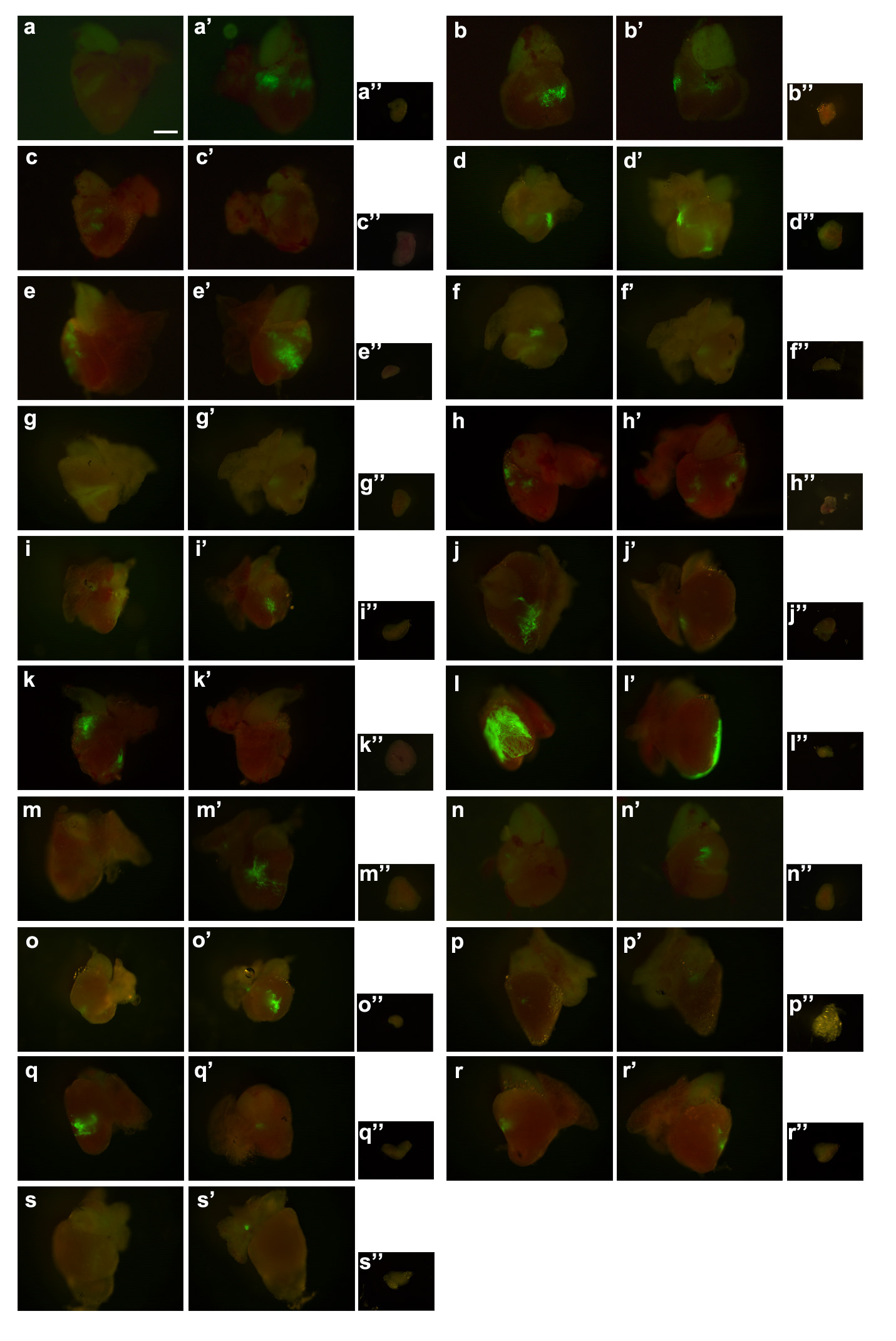
**Supplementary Fig. S4. Labeled CMs during heart regeneration.** 2dpf labeled zebrafish larvae were grown to adulthood (3-months-old) and subjected to ventricular amputation. Regenerating hearts were collected after 30dpa. 19 of the analyzed hearts had clones which did not contribute to the regenerated area. The ventral (**a-s**) and the dorsal (**a’-s’**) view of each of the 19 hearts at 30dpa are shown. (**a’’-s’’**) Excised portion of the tissue in the amputation procedure. dpa, days post amputation. Scale bar: 500µm


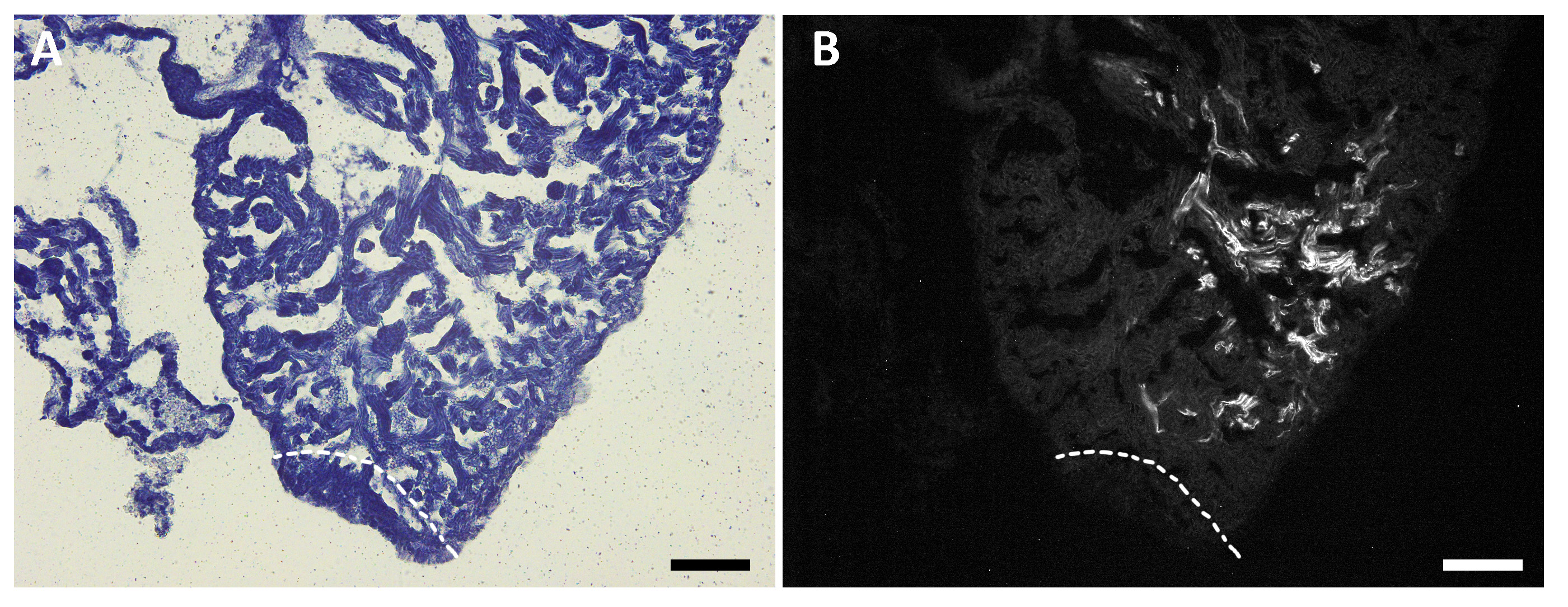


**Supplementary Fig. S5. Labeled CMs close to the injury site**. Sections processed for Sudan black staining showing the closest labeled cardiomyocytes to the injury site (**a,b**) in which GFP-positive cardiomyocytes did not contribute to the regenerated tissue. (**a**) Bright field image and (**b**) GFP positive clone. This heart corresponds to the regenerated heart in the panel q and q’ from the Supplementary figure S4. The amputation plane is indicated by dashed lines. Scale bars: 250μm
